# The Physical Fitness Level of College Students Before and After Web-Based Physical Education During the COVID-19 Pandemic

**DOI:** 10.3389/fped.2021.726712

**Published:** 2021-10-13

**Authors:** Wei Xia, Cai-hong Huang, Yu Guo, Min-gang Guo, Ming Hu, Jian Dai, Cheng-hu Deng

**Affiliations:** ^1^Department of Imaging Center, Wuhan Children's Hospital (Wuhan Maternal and Child Healthcare Hospital), Tongji Medical College, Huazhong University of Science and Technology, Wuhan, China; ^2^School of Physical Education, Hubei University of Technology, Wuhan, China; ^3^Department of Physical Education, Wuhan University of Technology, Wuhan, China

**Keywords:** COVID-19, college students, physical education, physical fitness, exercise

## Abstract

**Background:** The COVID-19 pandemic has been an emergency worldwide. Web-based physical education is a choice for college students to keep on their study. The aim of this study was to compare the data of physical fitness of college students before and after web-based physical education.

**Methods:** All the students of 2018 and 2019 in Wuhan University of Technology who had taken the web-based physical education class in 2020 were included in this study. The records of annual physical fitness tests of all the subjects in 2019 and 2020 which were carried out in September were reviewed, including weight, height, body mass index (BMI), vital capacity (VC), 50-m dash, sit-and-reach, standing long jump, male-specific pull-ups and 1,000-m race, and female-specific sit-ups and 800-m race.

**Results:** There were 24,112 male and 9,690 female records of physical fitness tests included in our study. The results of 11,219 male and 4,651 female students who completed both physical fitness tests in 2019 and 2020 were employed for Wilcoxon signed-rank test. Declined performance was observed on male 50-m dash by 0.1 s, male 1,000-m race by 14 s, and female 800-m race by 11 s. Notably, the percentage of male obesity, based on BMI, rose from 10.6 to 15.2% and 17.1 to 21.8% for male overweight; correspondingly, the percentage of male normal weight declined from 55.9 to 51.9% and 16.4 to 11.1% for male thinness. The trend of increasing BMI in males should be paid attention to. Improved results on vital capacity, sit-and-reach, standing long jump for both males and females, female 50-m dash, female sit-ups, and male pull-ups were observed in 2020. All the results of physical fitness tests were significantly different between 2019 and 2020 (*p* < 0.01) by Wilcoxon signed-rank test.

**Conclusions:** The changes of physical fitness tests before and after web-based physical education suggested that the focus should be placed on improvement for running tests through appropriate alternatives, such as fast running in place and shuttle run. In addition, the simple, convenient, and practical sport that require available equipment and little field should be considered for web-based physical education.

## Introduction

The outbreak of coronavirus disease 2019 (COVID-19), caused by a novel coronavirus named SARS-CoV-2, has affected the health of millions of people (1), which has been declared as a pandemic by the World Health Organization. To prevent the further spread of this severe infectious disease, quarantine was considered an effective method to protect the uninfected people from COVID-19 (2). As reported, the pandemic would last for a rather long period (3), as well as the quarantine. Education was disrupted by the closure of schools worldwide due to quarantine, with more than 990 million students involved reported by the United Nations Educational Scientific and Cultural Organization (4).

Although the quarantine was essential to prevent the further spread of COVID-19, it may also have limited the engagement of students in sufficient levels of physical activity. However, sufficient physical activity is essential not only for maintaining the physical well-being but also for keeping mental health in adolescents (5). Also, Chekroud's study has described that physical exercise was associated with lower mental health burden (6). Furthermore, Brooks's study has stated their concern on psychological impact of quarantine during this pandemic (7).

As insufficient physical activity was observed among adolescents in 146 counties by Cardon (8), how to restore the interrupted physical education for maintaining physical health was more essential than ever during this pandemic quarantine. Furthermore, Deng's study has described that mental health was significantly correlated with regular and sufficient exercise during this COVID-19 pandemic (9).

In order to keep physical and mental health of the adolescents, physical education was essential. As conventional education was not available, web-based education was carried out as an optional choice. Web-based physical education has been carried out in many universities and colleges during spring 2020 in Wuhan, but as an outdoor course, there were many challenges for web-based physical education.

So far as we knew, the effectiveness of newly developed web-based physical education has never been evaluated. In addition, as a newly developed style of physical education, it is difficult to evaluate the effectiveness of it comprehensively. Although physical education promoting physical activity and fitness has been long recognized (10, 11), the degree of physical education on improving physical fitness was still a controversial issue (12–14). However, physical fitness tests were still effective and quantitive tools for measuring effectiveness of physical education. In Bao's study, physical fitness tests were used to evaluate the effectiveness of mandatory physical education of the university students in aspects of body composition, cardiorespiratory endurance, flexibility, muscular strength, and muscular endurance (15).

Therefore, the primary purpose of our study was to describe the results of physical fitness tests before and after Web-Based Physical Education during the COVID-19 pandemic, in 2019 and 2020 separately. Also, the secondary purpose of our study is to compare the results of physical fitness tests in 2019 and 2020, though it could not be only attributed to web-based physical education, the changes of physical fitness results could give out the insufficient aspects of college students and the suggestion for web-based physical education in the future.

## Methods

### Study Population and Their Web-Based Physical Education

All the students of 2018 and 2019 in Wuhan University of Technology who had taken the web-based physical education class in 2020 were included in this study. The web-based physical education class was carried out once a week for 1 h and a half in 2020 spring. The time duration for warm-up, exercise, and relaxation was 15 min, 1 h, and 15 min, respectively. The exercise of web-based physical education class was chosen by students, including football, basketball, tennis, badminton, table tennis, gymnastics, and Chinese kung fu. Exercise for 1 h everyday was assigned as homework for all the students. This study was conducted in accordance with the Declaration of Helsinki and the ethical guidelines of medical research covering humans. This study was approved by institutional review board of Wuhan Children's Hospital (WHCH 2020029). Informed consents were disseminated by teachers to students and were provided by all the subjects.

### Measurements of Physical Fitness Tests

The records of annually physical fitness tests of all the subjects in 2019 and 2020 which were carried out in September were reviewed. All the tests were measured and recorded by Lingkang physical fitness test set (Jiangsu Lingkang Electronic Technology, Changzhou, China). The physical fitness test was evaluated by the same group of physical education teachers in Wuhan University of technology in 2019 and 2020. All the tests were carried out during 8:30 a.m.−12:00 noon and 1:30 p.m.−5:00 p.m. The height and weight of each student were obtained at the beginning of physical fitness test. Height was obtained as the length from the highest point of head to the heel of students standing straight. Weight was measured as the students weigh without heavy clothing and shoes on. The physical fitness level of each student was quantified by seven tests, with body mass index (BMI), vital capacity (VC), 50-m dash, sit-and-reach, and standing long jump for both males and females, while sit-ups and 800-m race for females only, pull-ups and 1,000-m race for males only. Body mass index (BMI): a ratio of weight and height of the body, calculated as the ratio of the weight of the body in kilograms to the square of the height in meters. VC: a measurement for lung function, defined as the maximum amount of air in milliliters that can be exhaled after a maximum inhalation. Fifty-meter dash: to assess the speed and acceleration, measured as a single sprint of 50 m with time recorded in seconds. Sit-and-reach: to evaluate the flexibility, measured from sitting with legs outstretched, students reached hands forward as far as possible with length over foot recorded in centimeters. Sit-ups (for females only): to assess the muscle endurance, measured from supine position with legs bended, students rose from lying to a sitting position as many as possible over 60 s, with number of correctly executed sit-ups recorded. Pull-ups (for males only): to evaluate muscle endurance, measured from hanging by hands from a horizontal bar, students pulled themselves up until the chin is at the level of the bar as many as possible, with number of correctly executed pull-ups recorded. Standing long jump: to assess explosive strength, measured from standing at a mark, students tried to jump as far as possible, with length recorded in centimeters. 800-m (for female)/1,000-m (for males) race: to assess the endurance, measured as a race of 800 m/1,000 m with time recorded in seconds.

### Statistical Analysis

The students who had not completed the physical fitness tests were excluded. The Kołmogorov–Smirnov test served to check normality. The descriptive statistics were used for all the results of physical fitness tests, including height (in cm), weight (in kg), BMI (in kg/m^2^), vital capacity (in milliliters), 50-m dash (in s), sit-and-reach (in cm), male-specific pull-ups and female-specific sit-ups (by count), standing long jump (in cm), and male-specific 1,000-m race and female-specific 800-m race (in s). The results of physical fitness tests were expressed as mean and 95% confidence interval as well as median and interquartile range, according to the normality test. Classification evaluation of physical fitness tests was according to the National Physical Health Standards for Students (revised in 2014) (16), BMI was evaluated according to Ko's study as lower BMI cutoff value has been suggested for Chinese (17), and Chi-square test was employed to compare the difference of physical fitness tests between 2019 and 2020. Mann–Whitney *U* test was used for comparing the differences of physical fitness tests of all included subjects between 2019 and 2020. The students who completed both physical fitness tests in 2019 and 2020 were included for Wilcoxon signed-rank test. The significant level was set at *p* < 0.01. SPSS Statistic 19.0 (IBM SPSS Statistics, New York, USA) was employed for statistical analysis.

## Results

### Subjects Characteristics

There were 9,371 students (male: 6,673, female: 2,698) in the 2018 grade and 9,338 (male: 6,795, female: 2,543) students in the 2019 grade. The students who had not completed the physical fitness tests were excluded. At last, there were 7,776 (male: 5,354, female: 2,422) in the 2018 grade and 8,591 (male: 6,191, female: 2,400) in the 2019 grade who had completed the physical fitness tests of 2019, 8,563 (male: 6,080, female: 2,483) in the 2018 grade, and 8,872 (male: 6,487, female: 2,385) in the 2019 grade who had completed the physical fitness tests of 2020 being included in this study. in total, there were 24,112 male and 9,690 female records of physical fitness tests being included in our study.

**Figure 1:**
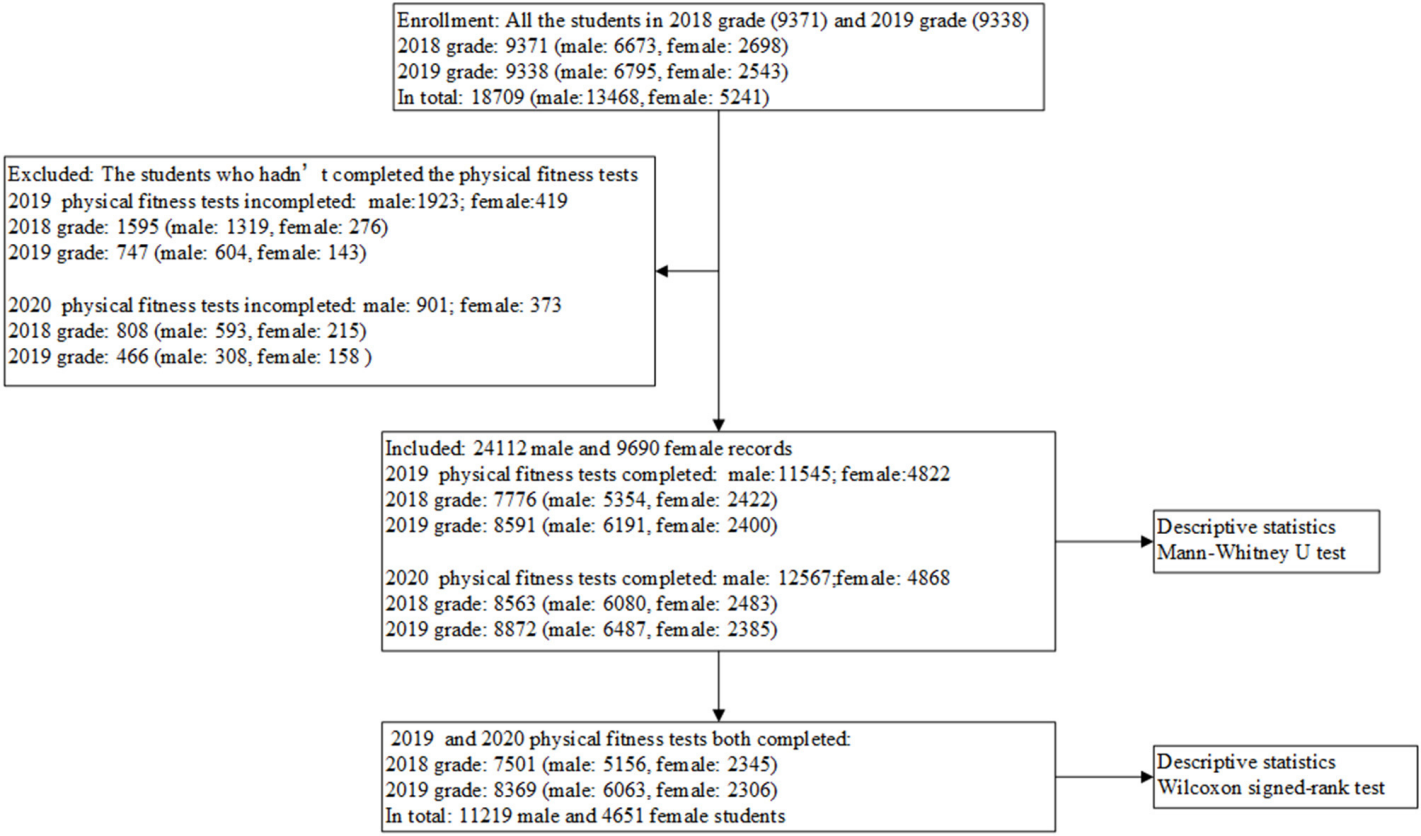
Flow chart of this study.

There were 11,219 male and 4,651 female students who completed both physical fitness tests in 2019 and 2020. The results of their all physical fitness tests were employed for Wilcoxon signed-rank test. The flow chart is shown in Figure 1.

### Descriptive Statistics and Mann–Whitney *U* Test for all Included Subjects

**Figure 2:**
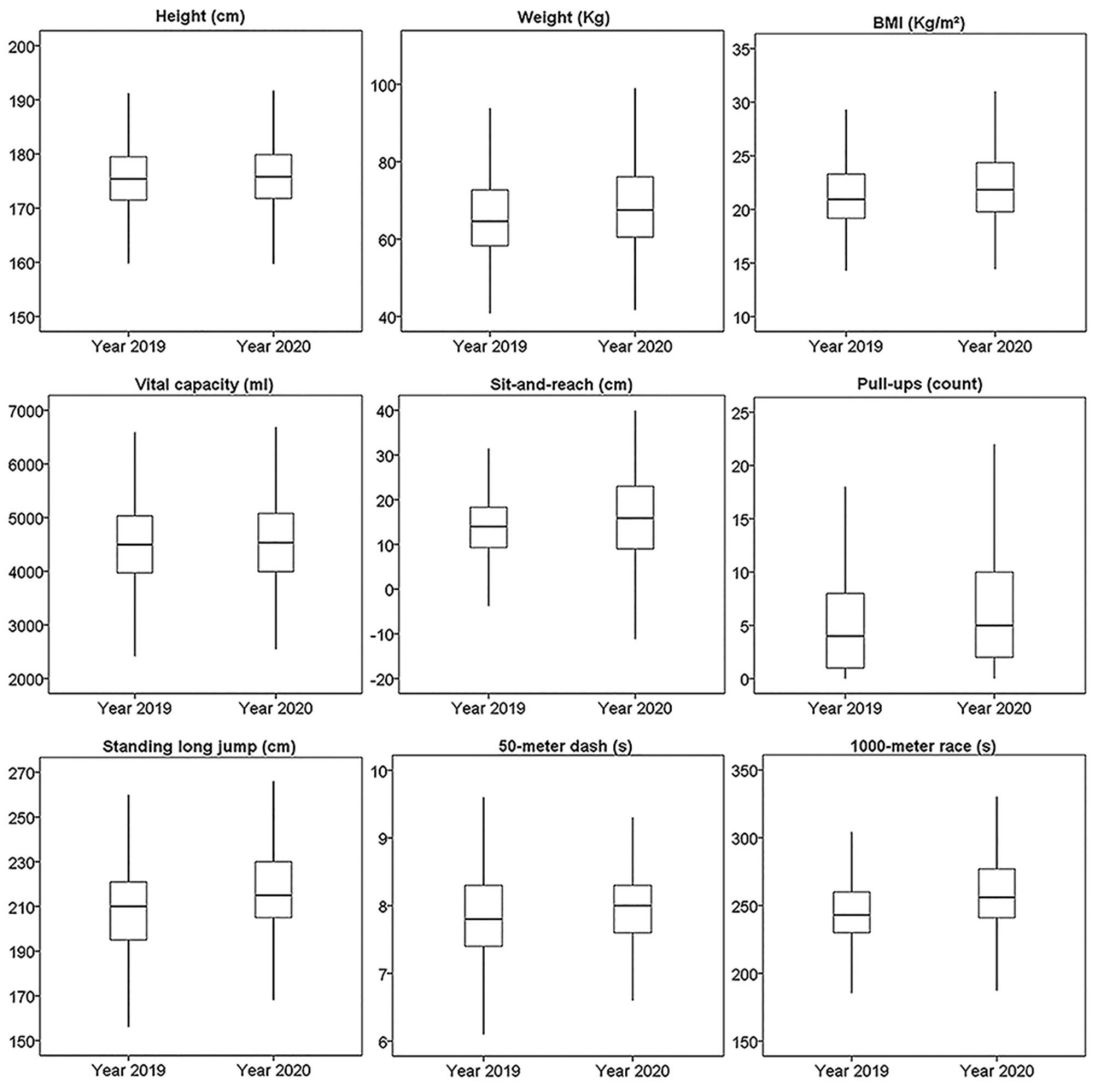
The boxplots of nine results of physical fitness tests from 24,112 male records. Middle line in the box represents the median. The upper and lower boundaries of the box represent upper and lower quartiles. The endpoints of whiskers represent upper and lower extremes.

**Figure 3:**
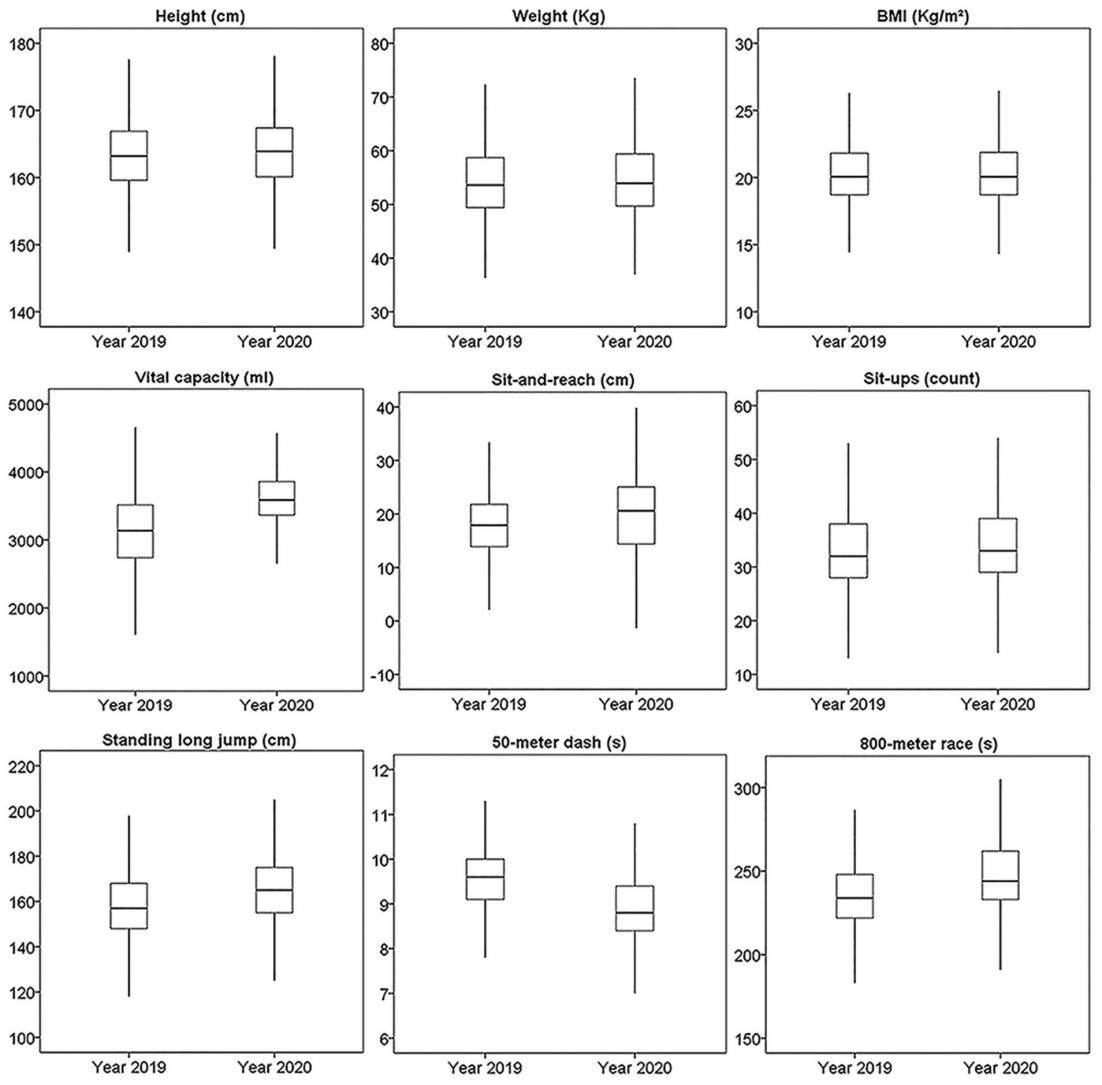
The boxplots of nine results of physical fitness tests from 9,690 female records. Middle line in the box represents the median. The upper and lower boundaries of the box represent upper and lower quartiles. The endpoints of whiskers represent upper and lower extremes.

As the data was not normally distributed, it was presented as median and interquartile range. The median and interquartile range of all the physical fitness tests were presented for males and females in Figures 2, 3, separately.

Classification evaluation of physical fitness tests from 24,112 male and 9,690 female records is shown in Table 1. Notably, the percentage of male obesity, based on BMI, rose from 10.6 to 15.2% and 17.1 to 21.8% for male overweight; correspondingly, the percentage of male normal weight declined from 55.9 to 51.9% and 16.4 to 11.1% for male thinness.

**Table 1:** Classification evaluation of physical fitness tests from 24,112 male and 9,690 female records.

	**Male in 2019**	**Male in 2020**	**Female in 2019**	**Female in 2020**
Age (year)	*p* < 0.001			*p* < 0.001
≤ 17	1,730 (15.0%)	112 (0.9%)	757 (15.7%)	48 (0.9%)
18	5,115 (44.3%)	1,715 (13.6%)	2,190 (45.4%)	701 (14.4%)
19	3,841 (33.3%)	5,457 (43.4%)	1,614 (33.5%)	2,203 (45.3%)
20	695 (6.0%)	4,292 (34.2%)	214 (4.4%)	1,629 (33.5%)
21	130 (1.1%)	789 (6.3%)	37 (0.8%)	235 (4.8%)
≥22	34 (0.3%)	202 (1.6%)	10 (0.2%)	52 (1.1%)
BMI (kg/m^2^)	*p* < 0.001			*p* = 0.559
Thinness (<18.5)	1,889 (16.4%)	1,397 (11.1%)	1,040 (21.6%)	1,012 (20.8%)
Normal (≥18.5 and <23)	6,452 (55.9%)	6,519 (51.9%)	3,090 (64.1%)	3,113 (63.9%)
Overweight (≥23 and <26)	1,979 (17.1%)	2,745 (21.8%)	503 (10.4%)	539 (11.1%)
Obesity (≥26)	1,225 (10.6%)	1,906 (15.2%)	189 (3.9%)	204 (4.2%)
VC (ml)	*p* < 0.001			*p* < 0.001
Excellent (≥4,800 for male, ≥3,300 for female)	4,084 (35.4%)	4,665 (37.1%)	1,879 (39.0%)	4,231 (86.9%)
Good (≥4,300 and <4,800 for male, ≥3,000 and <3,300 for female)	2,834 (24.5%)	3,522 (28.0%)	972 (20.2%)	593 (12.2%)
Pass (≥3,100 and <4,300 for male, ≥2,000 and <3,000 for female)	4,376 (37.9%)	4,288 (34.1%)	1,900 (39.4%)	44 (0.9%)
Fail (<3,100 for male, <2,000 for female)	251 (2.2%)	92 (0.8%)	71 (1.4%)	0 (0.0%)
50-m dash (s)	*p* < 0.001			*p* < 0.001
Excellent ( ≤ 6.9 for male, ≤ 7.7 for female)	736 (6.4%)	276 (2.2%)	45 (0.9%)	40 (0.8%)
Good ( ≤ 7.1 and >6.9 for male, ≤ 8.3 and >7.7 for female)	712 (6.2%)	459 (3.7%)	162 (3.4%)	905 (18.6%)
Pass ( ≤ 9.1 and >7.1 for male, ≤ 10.3 and >8.3 for female)	9,743 (84.4%)	11,714 (93.2%)	3,967 (82.3%)	3,334 (68.5%)
Fail (>9.1 for male, >10.3 for female)	354 (3.0%)	118 (0.9%)	648 (13.4%)	589 (12.1%)
Sit-and-reach (cm)	*p* < 0.001			*p* < 0.001
Excellent (≥21.3 for male, ≥22.2 for female)	1,581 (13.7%)	3,995 (31.8%)	1,039 (21.5%)	2,084 (42.8%)
Good (≥17.7 and <21.3 for male, ≥19 and <22.2 for female)	1,756 (15.2%)	1,555 (12.4%)	1,033 (21.4%)	850 (17.5%)
Pass (≥3.7 and <17.7 for male, ≥6 and <19 for female)	7,639 (66.2%)	6,406 (50.9%)	2,668 (55.3%)	1,797 (36.9%)
Fail (<3.7 for male, <6 for female)	569 (4.9%)	611 (4.9%)	82 (1.8%)	137 (2.8%)
Pull-ups/sit-ups (count)	*p* < 0.001			*p* < 0.001
Excellent (≥17 for male, ≥52 for female)	243 (2.1%)	189 (1.5%)	97 (2.1%)	112 (2.3%)
Good (≥15 and <17 for male, ≥46 and <52 for female)	224 (2.0%)	159 (1.3%)	267 (5.5%)	309 (6.3%)
Pass (≥10 and <15 for male, ≥26 and <46 for female)	1,861 (16.1%)	2,807 (22.3%)	3,814 (79.0%)	4,137 (85.0%)
Fail (<10 for male, <26 for female)	9,217 (79.8%)	9,412 (74.9%)	644 (13.4%)	310 (6.4%)
Standing long jump (cm)	*p* < 0.001			*p* < 0.001
Excellent (≥263 for male, ≥195 for female)	49 (0.4)	172 (1.4%)	122 (2.6%)	481 (9.9%)
Good (≥248 and <263 for male, ≥181 and <195 for female)	280 (2.4%)	708 (5.6%)	265 (5.5%)	384 (7.9%)
Pass (≥208 and <248 for male, ≥151 and <181 for female)	6,372 (55.2%)	8,231 (65.5%)	3,006 (62.3%)	3,439 (70.6%)
Fail (<208 for male, <151 for female)	4,844 (42.0%)	3,456 (27.5%)	1,429 (29.6)	564 (11.6%)
1,000-m/800-m race (s)	*p* < 0.001			*p* < 0.001
Excellent ( ≤ 207 for male, ≤ 210 for female)	382 (3.3%)	407 (3.2%)	408 (8.5%)	288 (5.9%)
Good ( ≤ 222 and >207 for male, ≤ 224 and >210 for female)	1,373 (11.9%)	547 (4.4%)	1,057 (21.9%)	472 (9.7%)
Pass ( ≤ 272 and >222 for male, ≤ 274 and >224 for female)	8,299 (71.9%)	7,885 (62.7%)	3,113 (64.6%)	3,458 (71.0%)
Fail (>272 for male, >274 for female)	1,491 (12.9%)	3,728 (29.7%)	244 (5.0%)	650 (13.4%)

*cm, centimeter; kg, kilogram; kg/m^2^, kilogram/meter^2^; ml, milliliter; s, second*.

All the results of physical fitness tests from male records were significantly different between 2019 and 2020 with *p* < 0.001. While except for BMI (*p* = 0.541), all the other results of physical fitness tests from female records were significantly different between 2019 and 2020 (*p* = 0.001 for weight; *p* < 0.001 for height, vital capacity, 50-m dash, sit-and-reach, sit-ups, standing long jump, and 800-m race).

### Descriptive Statistics and Wilcoxon Signed-Rank Test for Subjects Completed Both Physical Fitness Tests in 2019 and 2020

**Figure 4:**
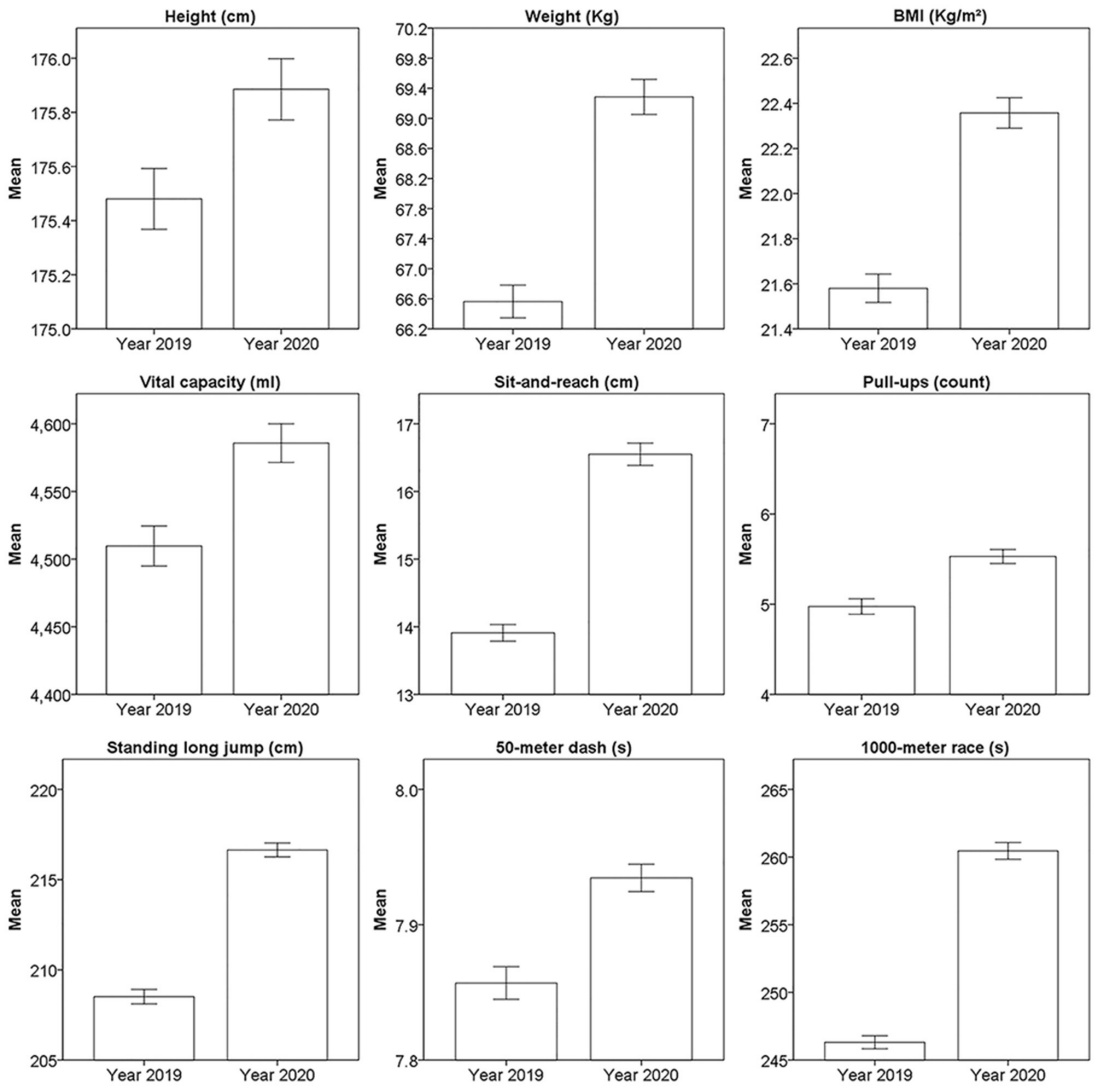
The mean and 95% confidence interval of physical fitness tests from 11,219 male students.

**Figure 5:**
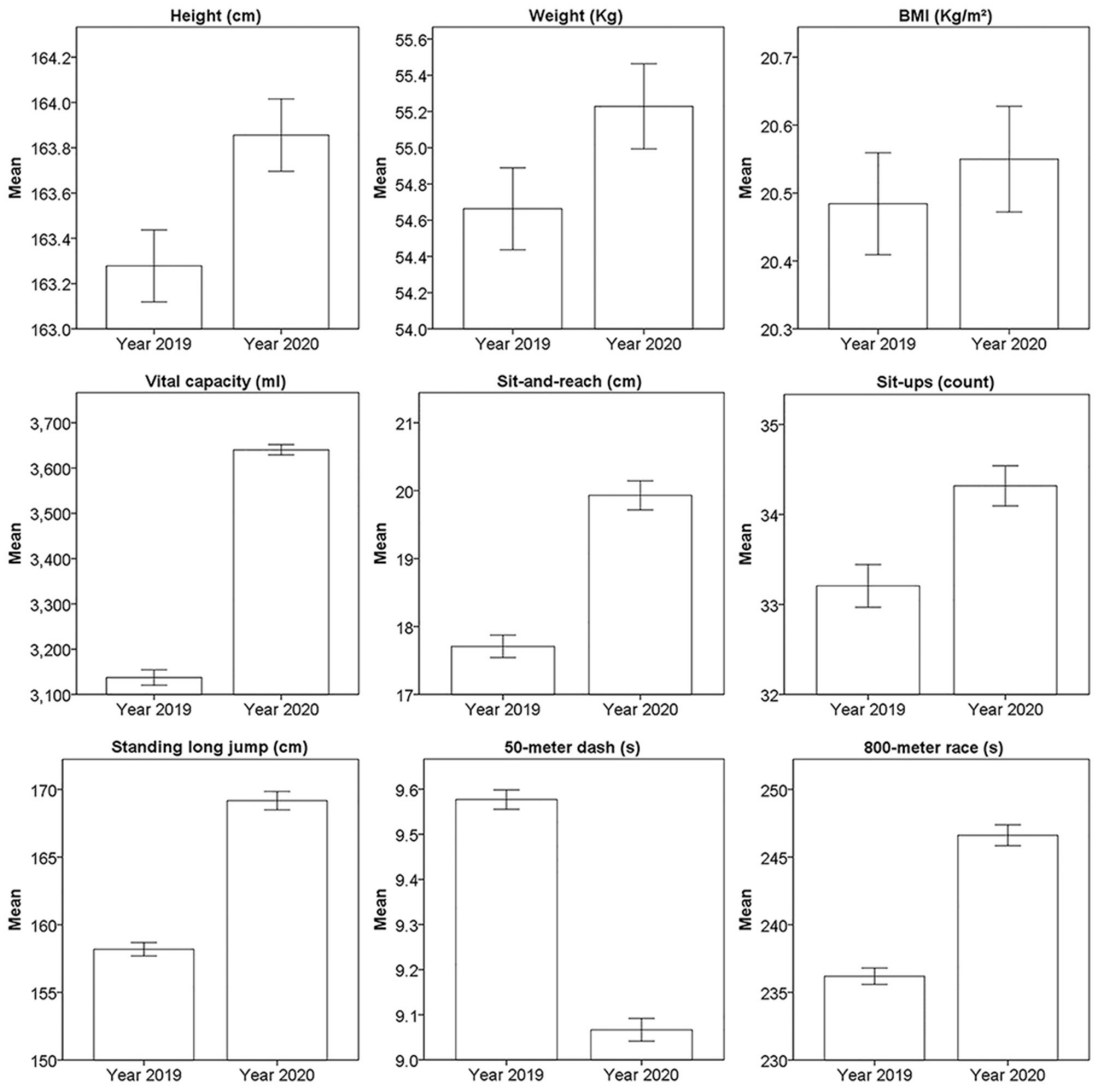
The mean and 95% confidence interval of physical fitness tests from 4,651 female students.

The mean and 95% confidence interval of physical fitness tests in 2019 and 2020 from 11,219 male and 4,651 female students are shown in Figures 4, 5 separately, all the subjects had completed both physical fitness tests in 2019 and 2020. The median, median of difference, and the *p*-value of 2019 and 2020 from these subjects is shown in Table 2. The *p-*value for all the results of physical fitness tests from 11,219 male students and 4,651 female students was <0.001. Notably, declined performance was observed on male 50-m dash by 0.1 s, male 1,000-m race by 14 s, and female 800-m race by 11 s.

**Table 2:** The comparison of physical fitness tests between 2019 and 2020 from 11,219 male students and 4,651 female students.

	**Male in 2019 (median, interquartile ranges)**	**Male in 2020 (median, interquartile ranges)**	**Median of difference, interquartile ranges**	***p*-Value**	**Female in 2019 (median, interquartile ranges)**	**Female in 2020 (median, interquartile ranges)**	**Median of difference, interquartile ranges**	***p*-Value**
Age (year)	18, 18–19	19, 19–20	1, 1–1	<0.001	18, 18–19	19, 19–20	1, 1–1	<0.001
Height (cm)	175.4, 171.5–179.5	175.8, 171.9–179.9	0.4, −0.2–1.0	<0.001	163.2, 159.6–1,660.9	163.8, 160.0–167.4	0.5, −0.2–1.3	<0.001
Weight (kg)	64.6, 58.2–72.5	67.3, 60.4–75.9	2.4, 0.2–5.2	<0.001	53.5, 49.4–58.6	53.9, 49.7–59.3	0.6, −1.2–2.4	<0.001
BMI (kg/m^2^)	20.9, 19.2–23.3	21.8, 19.7–24.3	0.7, 0.0–1.6	<0.001	20.1, 18.7–21.8	20.0, 18.7–21.9	0.1, −0.6–0.8	<0.001
VC (ml)	4,497, 3,970–5,028	4,537, 3,997–5,074	77, −278–432	<0.001	3,140, 2,742–3,515	3,588, 3,367–3,857	434, 63–928	<0.001
50-m dash (s)	7.8, 7.4–8.2	7.8, 7.6–8.3	0.1, −0.3–0.5	<0.001	9.6, 9.1–10.0	8.8, 8.4–9.4	−0.6, −1.1–0.0	<0.001
Sit-and-reach (cm)	14.0, 9.4–18.4	16.0, 9.2–23.1	1.6, −2.8–7.6	<0.001	18.0, 14.0–21.8	20.7, 14.6–25.2	1.5, −2.3–6.3	<0.001
Pull-ups/sit-ups (count)	4, 1–8	5, 2–10	0, −2–3	<0.001	33, 28–38	33, 29–39	1, −3–5	<0.001
Standing long jump (cm)	210, 195–222	215, 205–230	7, −3–19	<0.001	157, 148–168	165, 155–175	5, −3–15	<0.001
1,000-m/800-m race (s)	243, 229–259	256, 241–276	14, 0–29	<0.001	234, 222–248	244, 233–262	11, 0–23	<0.001

*cm, centimeter; kg, kilogram; kg/m^2^, kilogram/meter^2^; ml, milliliter; s, second*.

## Discussion

Under the circumstance of quarantine, the significant reduction of physical condition was first observed in professional athletes (18–21). However, the physical fitness of adolescents should be paid special attention to because of the reduction of physical activity reported recently (22–24). Considering the protection of physical activity on physical and mental health, Chen recommended regular physical activity and routinely exercising in a safe home environment (25). Furthermore, physical education teacher could improve the physical fitness more significantly than generalist teacher reported by Starc (26), specialist physical education was irreplaceable. Therefore, web-based physical education is a safe and new style for college students under this shutdown. However, the change of physical fitness before and after the web-based physical education has never been described so far as we know. The physical fitness tests in our study could represent the body composition (represented by height, weight, and BMI), cardiorespiratory function (represented by vital capacity and 1,000/800-m race), flexibility (represented by sit-and-reach), strength of muscle (represented by standing long jump and 50-m dash), and endurance of muscle (represented by pull-ups for male/sit-ups for female and 1,000/800-m race). The different change of result from each test would reflect the disadvantage of physical fitness and the focus of web-based teaching in the future.

As to the body composition, we would like to discuss the height, weight and BMI together, as the students were still growing adolescents. The height of both male and female student increased in 2020 along with the time because of the growing development, as well as the weight. According to the suggested BMI for Chinese, 18.5 ≤ BMI <23 was considered normal (17). The percentage of male obesity rose from 10.6 to 15.2% and 17.1 to 21.8% for male overweight; correspondingly, the percentage of male normal weight declined from 55.9 to 51.9% and 16.4 to 11.1% for male thinness. Significantly increased percentage of male obesity and overweight in 2020 meant the increase of weight gain exceeding the height, which may suggest insufficient exercise or excessive intake of food. As obesity has been considered a chronic relapsing disease process now, healthy life style including proper exercise is essential for keeping normal BMI (27). Hallal's team has reported that males were more positive in exercise than females (28), and their engagement in sports may be interrupted more severely by quarantine which resulted in insufficient exercise. Although the BMI of majority of males was still in normal range, sufficient exercise should be restored in order to prevent obesity and its detrimental effects.

Quanjer's work has reported the reference values of vital capacity which is an essential index for lung function (18). The interquartile range of vital capacity in our work basically fell in to the reference values by Quanjer's team. As no relationship between BMI and vital capacity was found by previous reports (20, 24), the change of vital capacity could not be explained only by the natural growing development. In our study, significant difference was noticed on vital capacity between 2019 and 2020, especially for female. According to Dugral's study, exercised young adults exhibited better vital capacity, especially for females. In addition, they suggested that lack of exercise significantly worsened the lung function (29). Considering our results on vital capacity, a certain amount of exercise should have been carried out by the subjects in our study. At least, absence of physical activity was not observed in our study because of improved vital capacity.

As previous studies have reported, quarantine has already negatively influenced the physical activity level of youngsters and adolescents in Norway and Germany (30, 31). However, significant improvement was observed in sit-and-reach, male-specific pull-ups, female-specific sit-ups, and standing long jump in our study, which represented flexibility, muscular strength, and muscular endurance separately. Under the circumstance of quarantine, the physical activity mentioned above could still be carried out readily without being influenced by restriction of field and equipment, which was in accordance with the report of protection factor for maintaining physical activity level by use of home exercise equipment during this shutdown from Fearnbach's group (32).

As reported by Scheer, the events that limited by distance and time was significantly decreased during this pandemic, and they suggested that it was impossible that running events could return to pre-pandemic levels soon (33). Negative influence on running tests was observed on male 50-m dash and 1,000-m/800-m race in our study as well. Under the circumstance of shutdown, we supposed that inadequate practice on running due to unavailable field led to worse results in 2020. However, the improvement on female 50-m dash suggested that muscular strength could be improved even under this quarantine. Though the reason for this improvement was still not clear, we supposed it may be related to relatively stable BMI in females and appropriate alternative exercise for 50-m dash on muscular strength, according to previous reports by Chen (24). Further study would be carried out to search for alternative exercises not only on muscular strength but also for endurance of muscle.

Under this circumstance of shutdown, health-related life style should be restored, especially for adolescents (13, 34). As physical education teacher plays an irreplaceable role in the education, and web-based physical education was the new and safe choice for college students, we would like to know the focus of web-based physical education under this pandemic. The physical fitness tests before and after the web-based physical education could reflect the changes of physical condition in college students during this shutdown. Although there were many confounding factors, such as family socioeconomic status, local neighborhood, accessible sport facility, and exercise habit, physical education still played an important role on physical fitness in college students (12). Negative influence has been observed on BMI, male 50-m dash and 1,000-m/800-m race, while improvement on all the other tests was surprising. We could draw the conclusion that the development of physical fitness was unbalanced. The improved results on cardiorespiratory function (represented by vital capacity), flexibility (represented by sit-and-reach), strength of muscle (represented by standing long jump), and endurance of muscle (represented by pull-ups for male/sit-ups for female) in 2020 may be related to their simple, convenient, and practical implement. While the worse results on running tests may be related to restricted sports facility. According to Chen's and de Sa-Caputo's suggestion, the sports that only required available equipment and little field would be more recommendable, such as sit-ups, pull-ups, sit-and-reach, and Tai Ji Quan (23, 25). We suggested that web-based physical education should choose sports which were more convenient and implementable. In addition, the exercise as the alternatives for 50-m dash and 800/1,000-m race should be taken into consideration for web-based physical education, such as fast running in place and shuttle run which just needed little field. Furthermore, we could gain experience from other courses and obtain support from emerging fields, such as virtual reality (35).

There were several limitations in our study. First, as the sudden outbreak of COVID-19 pandemic, prospective study was unavailable under this circumstance, and the subjects in our study may not represent the sample for Chinese students. Second, the confounding factors were not controlled in our study, such as physical education class selection, completion of homework, location of the students, social economic status, mental status, psychometric evaluation, sleep disorders, etc.; the results of physical fitness could not be explained as the effect of web-based physical education simply, but the latter one should be one of the important factors of the former one, according to previous studies (11, 26, 36).

## Conclusions

The declined performance on male 50-m dash, male 1,000-m race, and female 800-m race was observed in 2020 after web-based physical education. The trend of increasing BMI in males should be paid attention to. While improved results on vital capacity, sit-and-reach, standing long jump for both males and females, female 50-m dash, female sit-ups, and male pull-ups were obtained in 2020. The changes of physical fitness tests before and after web-based physical education suggested that the focus should be placed on improvement for running tests through appropriate alternatives, such as fast running in place and shuttle run.

## Data Availability Statement

The raw data supporting the conclusions of this article will be made available by the authors, without undue reservation.

## Ethics Statement

The studies involving human participants were reviewed and approved by Institutional Review Board of Wuhan Children's Hospital (WHCH 2020029). The patients/participants provided their written informed consent to participate in this study.

## Author Contributions

WX, CH, YG, MG, and CD: conception and design of the study. WX, CH, YG, MG, MH, JD, and CD: data collection. WX, CH, YG, MG, MH, JD, and CD: analysis and interpretation of data. WX and CD: drafting the article. WX, CH, and CD: literature review. All authors critically revising the article, final approval of the manuscript, and have verified the collected data.

## Funding

This research was supported by a grant from Hubei Province health and family planning scientific research project (Grant Number: WJ2018H0160), a grant from the Science and Technology Department of Hubei Province (Grant Number: 2020CFB710), a grant from the Wuhan University of Technology Teaching Reform Research Project (Grant Number: 2021190), and a grant from the Wuhan Children's Hospital Foundation (Grant Number: 2020FE002).

## Conflict of Interest

The authors declare that the research was conducted in the absence of any commercial or financial relationships that could be construed as a potential conflict of interest.

## Publisher's Note

All claims expressed in this article are solely those of the authors and do not necessarily represent those of their affiliated organizations, or those of the publisher, the editors and the reviewers. Any product that may be evaluated in this article, or claim that may be made by its manufacturer, is not guaranteed or endorsed by the publisher.

## Abbreviations

BMI: body mass index
VC: vital capacity.
